# F2-layer height of the peak electron density (hmF2) dataset employed in Inferring Vertical Plasma Drift – Data of Best fit

**DOI:** 10.1016/j.dib.2018.04.141

**Published:** 2018-05-05

**Authors:** B.O. Adebesin, J.O. Adeniyi

**Affiliations:** Space Weather Group, Department of Physical Sciences, Landmark University, PMB 1001 Omu-Aran, Kwara State, Nigeria

**Keywords:** African equatorial region, Digital portable sounder, Vertical plasma drift, Pre-noon, hmF2, F2-layer

## Abstract

In this data article, analysis of the height of the peak electron density (*hmF2*) data, used to compute the vertical plasma drift (*Vz*) velocities during year 2010, was reported. The station of focus is Ilorin, a station in the African equatorial region. The *hmF2* data used for the *Vz* computation was obtained from the Global Ionospheric Radio Observatory (GIRO) network of ionosondes, using the Digital Portable Sounder erected at the Equatorial Ionospheric Observatory of the University of Ilorin, Nigeria. *Vz* velocities were determined from the time rate of change of *hmF2*. Four categories of *hmF2* data intervals for determining the drift were analysed and compared for reliable computation of *Vz*. This are the measured 15-minute, the calculated 30-minute, the calculated 60-minute, and the directly selected 1-hour interval datasets. The calculated 60-minute interval data was found more reliable than others, satisfying the three significant events that characterized vertical drift observations. These are the evening time pre-reversal enhancement, the daytime pre-noon upward drift, and the nighttime downward reversal periods. The observations from this data will help Space weather scientists and researchers in identifying the best fit of *hmF2* data in the computation of drift velocity. The original work which has been published in Adebesin et al. (2013) [1] had made use of this calculated 60-minute interval *hmF2* data, but the process/procedures of its selection as the best fit of data interval was not explained in that work.

**Specifications Table**

**Table:** 

Subject area	Aeronomy and Space Weather environment
More specific subject area	Ionospheric Equatorial Electrodynamics
Type of data	Tables and figures
How data was acquired	Secondary Data
Data format	Raw, filtered, processed, and analyzed
Experimental factors	Data obtained from Global Ionospheric Radio Observatory (GIRO) network of ionosondes
Experimental features	Computational Analysis, and presented on monthly hourly averages
Data source location	Data obtained from Global Ionospheric Radio Observatory (GIRO) network of ionosondes. Data is for Ilorin (Geographic latitude 8.50°N, longitude 4.68°E), Nigeria, West Africa.
Data accessibility	The processed monthly hourly averaged data are available within this paper.

**Value of the data**

• The hmF2 data shown here will be useful for computing the vertical plasma drift velocity, especially in the African equatorial sector where direct vertical drift measurement technique is not available.
• The data can be used to study the Ionospheric electrodynamics in the African sector, and add to the global understanding of Space Weather environment.
• The data will be useful for Space Weather researchers and Telecommunication/radio propagation experts, in ionospheric irregularities nowcasting, forecasting, and possible mitigation purposes.

## 1 Data

### 1.1 Source of data

**Fig. 1:**
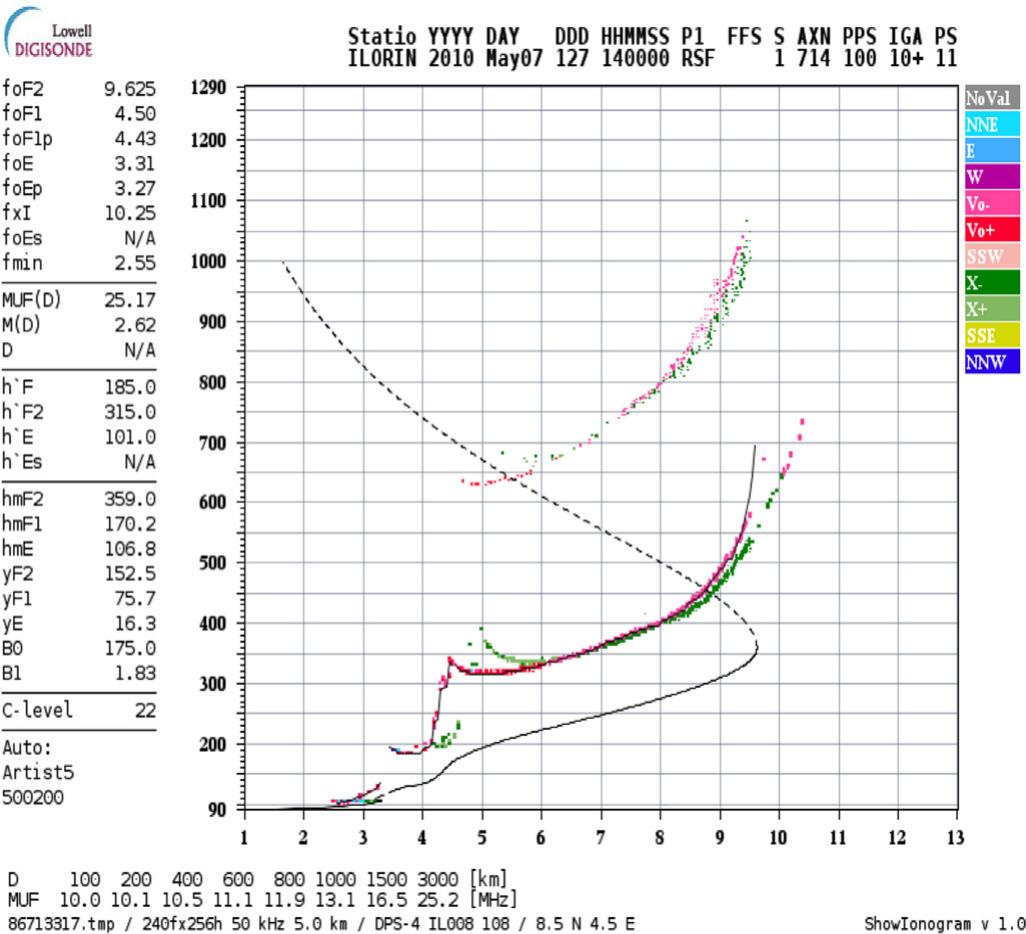
Typical ionogram. Observation is for 2010 over Ilorin ionosphere at 14:00 hour for 07 May. Other ionospheric parameters that can be obtained from the digisonde are displayed on the Left Hand Side with corresponding values.

**Fig. 2:**
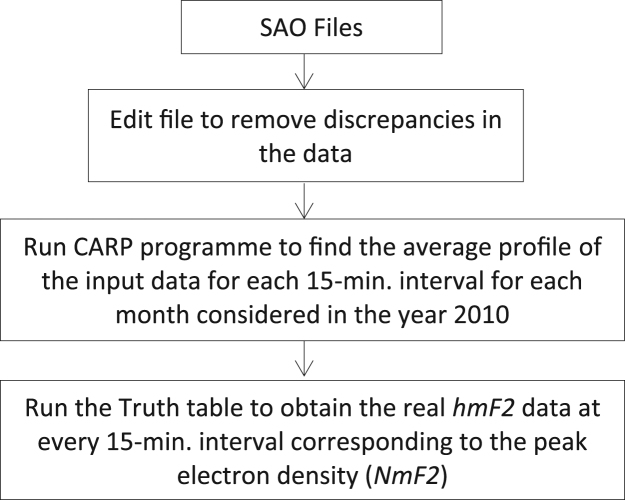
Process involved in obtaining hmF2 values using the CARP program.

The ionospheric *hmF2* data for this article were obtained from the Global Ionospheric Radio Observatory (GIRO) network of ionosondes, using the digital ionogram database (DIDBase), obtained from http://ulcar.uml.edu/DIDBase/. The data was for the Digital Portable Sounder (DPS-4.2 version) erected at the Equatorial Ionospheric Observatory of the University of Ilorin, Ilorin (Geographic lat. 8.50°N, long. 4.68°E), Nigeria. The raw data is an ionogram (e.g. Fig. 1) automatically scaled and processed. The output of the process is in the Standard Archiving Output (SAO) format of 15-minute interval. The raw SAO data format was then processed by the use of the Calculated Average Representative Profile (CARP) program developed by [2] – the process known as ionogram inversion. Fig. 2 highlights the processes involved in calculating the average monthly profiles for the *hmF2* observation from the CARP program at every 15-minute interval.

### 1.2 hmF2 data treatment

After the hmF2 data was obtained at every 15 min interval, there is the question of whether the 15 min interval hmF2 data will be good enough to infer the values for the vertical plasma drift velocities – in terms of aligning with the pattern and allowed/permitted drift velocity magnitude. Generally, *Vz* is usually categorized in three segments. These are (i) the evening time pre-reversal enhancement - PRE, often around 18:00–20:00 LT, (ii) the daytime pre-noon period (usually around 10:00–11:00 LT) at which time the drift is upward, and (iii) the nighttime reversal period, during when the drift has reversed downward ([3]). Subsequently, four different types of window interval of *hmF2* were tested, and the best of it was selected for the computation of Vz. The four types are (i) the measured 15-minute interval *hmF2* (*hmF2*_*15*_), (ii) the calculated 30-minute interval hmF2 (*hmF2*_*30*_), (iii) the calculated 60-minute interval hmF2 (*hmF2*_*60*_), and (iv) the direct 1-hour interval hmF2 (*hmF2*_*1HR*_) obtained directly at every one hour interval. Table 1 highlights the procedure involved in analyzing the four classes of *hmF2* datasets. The footnote of the table describes the mathematical expressions for each class of dataset. While *hmF2*_*15*_ is the original time interval, both the *hmF2*_*30*_ and *hmF2*_*60*_ were generated. Further, the *hmF2*_*1HR*_ was picked directly at every hour interval of *hmF2*_*15*_. (i.e. S1 = P1 at 0:00 LT, while S2 = P5 at 1:00 LT). LT is the local time, and it is measured in hour.

**Table 1:** Procedure for data derivation for the *hmF2*_*15*_, *hmF2*_*30*_, *hmF2*_*60*_, and *hmF2*_*1HR*_.

**HOUR (LT)**	**15 min interval hmF2 (km)*****(hmF2***_***15***_***)***	**30 min interval hmF2 (km)*****(hmF2***_***30***_***)***	**60 min interval hmF2 (km)*****(hmF2***_***60***_***)***	**Direct 1 h interval hmF2 (km)*****(hmF2***_***1HR***_***)***
0:00	P1	Q1	R1	S1
0:15	P2			
0:30	P3	Q2		
0:45	P4			
1:00	P5	Q3	R2	S2
1:15	P6			
1:30	P7	Q4		
1:45	P8			
2:00	P9	Q5	R3	S3
2:15	P10			
2:30	P11	Q6		
2:45	P12			
3:00	P13	Q7	R4	S4
3:15	P14			
3:30	P15	Q8		
3:45	P16			
4:00	P17	Q9	R5	S5
**…**	…	…	…	…

Q2=Mean (P2:P4); Q3=Mean (P4:P6); Q4=Mean (P6:P8); etc.

R2=Mean (P3:P7); R3=Mean (P7:P11); R4=Mean (P11:P15); etc.

S1= P1; S2 = P5; S3 = P9; etc.

**Fig. 3:**
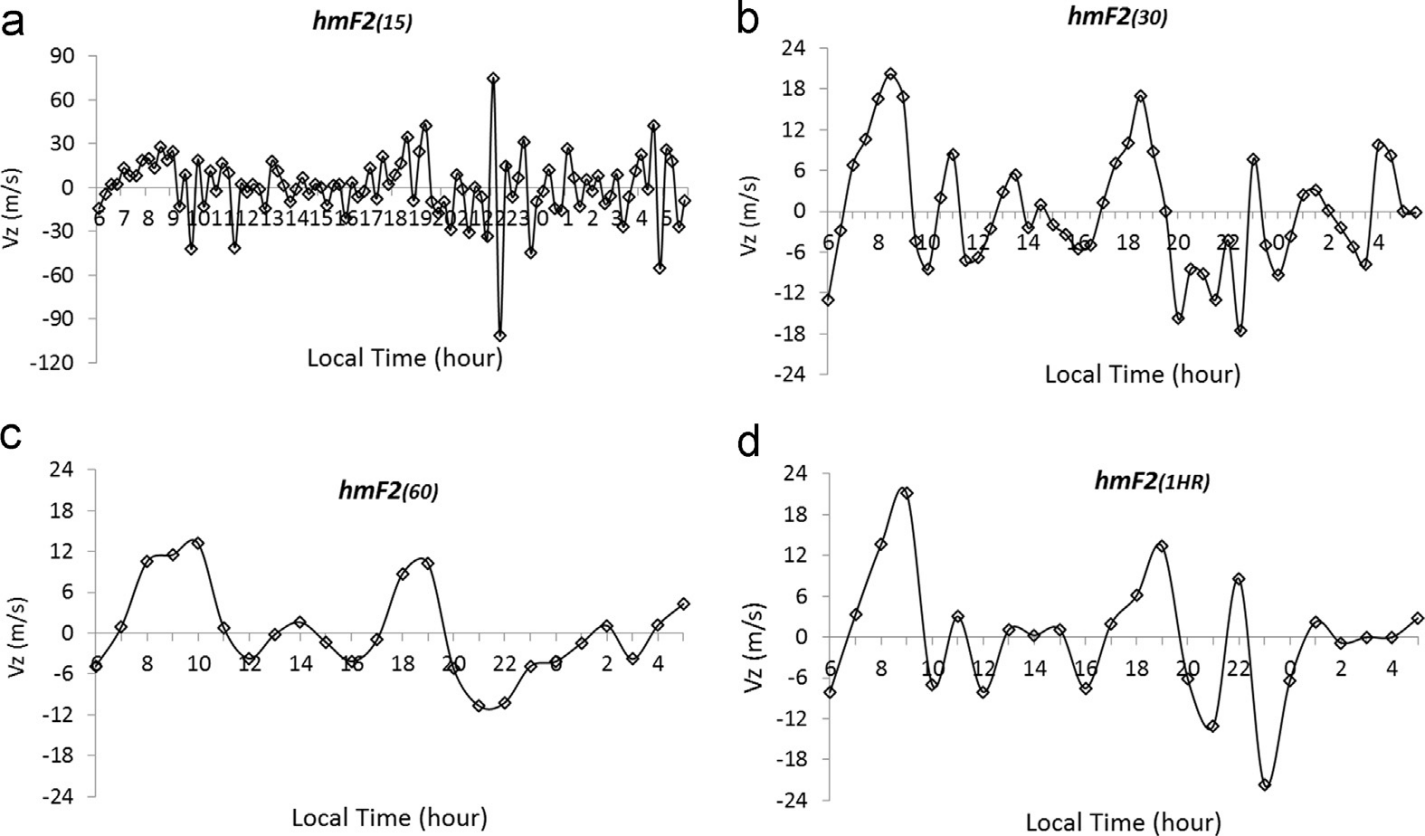
Plot of Vz inferred from hmF2 for March 2010 over Ilorin F2 ionosphere for (a) 15-min hmF2 interval (b) 30-min hmF2 interval (c) 60-min hmF2, and (d) 1-h hmF2 direct picking interval. (Both the 30-min and 60-min Intervals were generated from the series of 15-min Interval data as shown in Table 1).

A sample plot of *Vz* was then employed for each type of hmF2 interval data using its time rate of change. This is revealed in Fig. 3. Obviously, drift velocity inferred from *hmF2*_*15*_ data do not meet with up with the three features that characterizes *Vz* pattern. It also overestimated the evening time PRE magnitude to a higher percentage, well above the permitted magnitude. We are then left with *hmF2*_*30*_, *hmF2*_*60*_, and *hmF2*_*1HR*_ to pick from, as the best interval data in the computation of drift velocity.

**Fig. 4:**
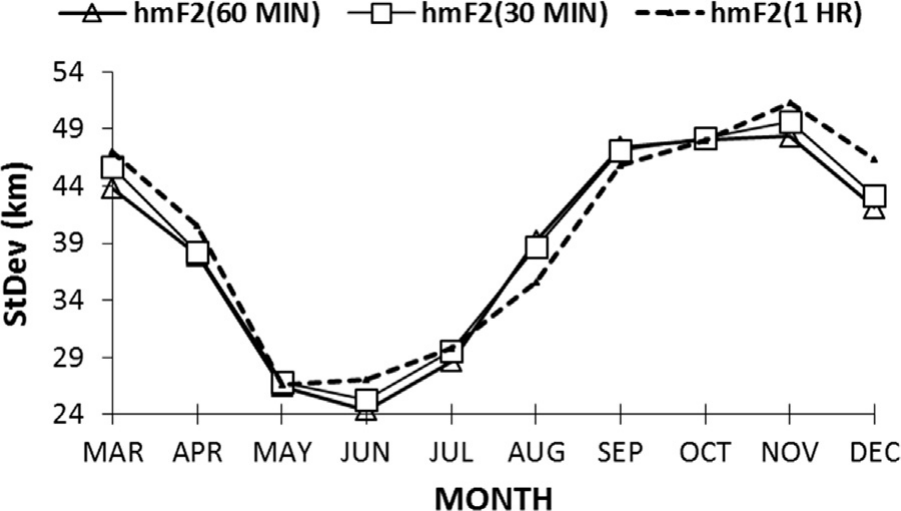
Monthly average Standard deviation (Stdev) for *hmF2*_*30*_, *hmF2*_*60*_, and *hmF2*_*1HR*_ for the year 2010.

Both the *hmF2*_*30*_ are *hmF2*_*60*_ had the advantage that all the data were used during the computation process (Table 1). The remaining three datasets were thereafter subjected to statistical analysis. Highlighted in Table 2 are the hmF2 monthly mean statistical data for the three remaining data-set intervals. Data are not available for the months of January and February, as the Digital Portable Sounder was erected at the Ilorin Observatory in March 2010. Reading from the relative standard deviation and the relative standard error values for the *hmF2*_*30*_, *hmF2*_*60*_, and *hmF2*_*1HR*_, *hmF2*_*60*_ was observed to be more reliable in the computation of Vz (owning to its lowest relative standard error values in most of the months). The standard deviation plot, in Fig. 4, for the *hmF2*_*30*_, *hmF2*_*60*_, and *hmF2*_*1HR*_ also showed that the 60-minute interval data is better than the other two; though the standard deviation plots revealed similar pattern. This conditions now led us to the use of the *hmF2*_*60*_ as our hmF2 data in the computation of the drift velocity. The data is presented in Table 3, which is the hourly monthly average; and the data of best fit for Vz.

**Table 2:** Monthly mean statistical data of hmF2 showing the variability in the three data-set Intervals of *hmF2*_*30*_, *hmF2*_*60*_, and *hmF2*_*1HR*_. The most statistically reliable for each month is tagged by a.

**Month**	**hmF2**	**Mean (km)**	**Variance (km)**	**Standard deviation (km)**	**Relative standard deviation**	**Standard error (km)**	**Relative standard error**
March	*hmF2*_*60*_	311	1920	43.81	0.1410	8.94	0.0288^a^
	*hmF2*_*30*_	313	2087	45.68	0.1459	9.33	0.0298
	*hmF2*_*1HR*_	316	2208	46.99	0.1483	9.59	0.0303
April	*hmF2*_*60*_	302	1439	37.93	0.1257	7.74	0.0257^a^
	*hmF2*_*30*_	303	1461	38.23	0.1260	7.80	0.0258
	*hmF2*_*1HR*_	309	1643	40.53	0.1310	8.27	0.0267
May	*hmF2*_*60*_	287	701	26.48	0.0923	5.40	0.0188^a^
	*hmF2*_*30*_	288	717	26.78	0.0929	5.56	0.0190
	*hmF2*_*1HR*_	294	710	26.64	0.0924	5.44	0.0189
June	*hmF2*_*60*_	285	594	24.37	0.0854	4.97	0.0174^a^
	*hmF2*_*30*_	286	642	25.34	0.0885	5.17	0.0181
	*hmF2*_*1HR*_	290	730	27.01	0.0933	5.51	0.0190
July	*hmF2*_*60*_	292	822	28.67	0.0976	5.85	0.0199^a^
	*hmF2*_*30*_	293	875	29.58	0.1008	6.04	0.0206
	*hmF2*_*1HR*_	294	890	29.83	0.1020	6.09	0.0208
August	*hmF2*_*60*_	305	1538	39.22	0.1286	8.01	0.0262
	*hmF2*_*30*_	306	1494	38.66	0.1264	7.89	0.0258
	*hmF2*_*1HR*_	299	1264	35.56	0.1190	7.26	0.0243^a^
September	*hmF2*_*60*_	305	2248	47.42	0.1555	9.68	0.0317
	*hmF2*_*30*_	306	2218	47.10	0.1535	9.61	0.0313
	*hmF2*_*1HR*_	307	2098	45.80	0.1487	9.35	0.0304^a^
October	*hmF2*_*60*_	316	2308	48.05	0.1520	9.80	0.0310^a^
	*hmF2*_*30*_	318	2329	48.26	0.1520	9.85	0.0311
	*hmF2*_*1HR*_	314	2303	47.99	0.1528	9.80	0.0311
November	*hmF2*_*60*_	320	2336	48.34	0.1510	9.87	0.0308^a^
	*hmF2*_*30*_	322	2463	49.63	0.1541	10.13	0.0314
	*hmF2*_*1HR*_	319	2635	51.33	0.1606	10.48	0.0328
December	*hmF2*_*60*_	324	1774	42.12	0.1299	8.60	0.0265^a^
	*hmF2*_*30*_	326	1862	43.15	0.1323	8.81	0.0270
	*hmF2*_*1HR*_	326	2146	46.33	0.1422	9.46	0.0290

a most reliable dataset interval for each month

**Table 3:** Hourly Monthly average hmF2 data used (The 60 min interval data – Data of best fit).

**TIME**	**hmF2 (km)**									
	**Mar.**	**Apr.**	**May**	**Jun.**	**Jul.**	**Aug.**	**Sep.**	**Oct.**	**Nov.**	**Dec.**
00:00	266	272	278	268	281	280	269	280	274	285
01:00	260	271	259	266	292	288	262	286	274	279
02:00	264	264	263	265	286	281	253	291	272	298
03:00	251	258	274	263	282	263	249	280	279	293
04:00	255	268	284	270	266	254	251	259	263	262
05:00	271	271	276	263	259	266	259	253	264	260
06:00	253	244	243	244	244	245	247	247	265	263
07:00	257	249	249	260	248	250	244	255	265	273
08:00	305	275	264	280	261	264	268	286	307	302
09:00	305	275	264	280	261	264	268	286	307	302
10:00	353	344	301	322	310	332	339	364	363	372
11:00	356	350	308	334	322	348	340	361	356	357
12:00	342	340	318	323	323	352	346	353	352	350
13:00	342	342	327	315	324	360	352	354	347	343
14:00	347	346	335	322	332	355	359	348	350	355
15:00	343	341	327	313	331	352	363	354	351	370
16:00	328	327	322	301	327	338	350	372	351	364
17:00	324	326	304	281	324	331	344	370	361	345
18:00	356	340	300	281	323	334	361	380	390	362
19:00	392	355	296	292	319	343	379	393	418	390
20:00	374	329	285	286	301	335	350	354	391	383
21:00	335	303	269	278	279	300	309	314	325	354
22:00	298	276	261	269	260	295	286	274	286	319
23:00	281	275	280	270	264	286	271	266	269	300

The various statistical instruments used in Table 2 are defined in Eqs. (1), (2), (3), (4), (5), (6) as follows:

(1) $$Mean,\bar{X}=\frac{\sum_{i=1}^{n}X_{i}}{n}$$

where the summation $X_{i}$ is the sum of the hmF2 values at *hmF2*_*30*_, *hmF2*_*60*_, and *hmF2*_*1HR*_ respectively, for different months, and n is the number of points (hours) considered.

(2) $$Variance,\sigma^{2}=\frac{\sum_{i=1}^{n}{\left(X_{i}-\bar{X}\right)}^{2}}{n}$$

(3) $$Standard\;Deviation,\sigma =\sqrt{\frac{\sum_{i=1}^{n}{\left(X_{i}-\bar{X}\right)}^{2}}{n}}$$

(4) $$Relative\;Standard\;Deviation,\sigma_{R}=\frac{\sigma }{\bar{X}}$$

(5) $$Standard\;Error,\sigma_{\bar{x}}=\frac{\sigma }{\sqrt{n}}$$

(6) $$Relative\;Standard\;Error,\sigma_{\bar{r}}=\frac{\sigma_{\bar{x}}}{\bar{X}}$$

## 2 Materials and methods

From the monthly hourly mean values of hmF2 values presented in Table 3, vertical E x B plasma drift velocities were determined by measuring the time rate of change of F2 real heights using the mathematical expressions in Eq. (7)

(7) $$\mathrm{Vz}=\frac{d(\mathrm{hmF}2)}{\mathrm{dt}}$$

The months were then classified into different seasons viz: March equinox (February, March April), June solstice (May, June, July), September equinox (August, September, October) and December solstice (November, December, January) (e.g. [4]) for further drift analysis.

## References

[1] Adebesin B.O., Adeniyi J.O., Adimula I.A., Reinisch B.W. (2013). Equatorial vertical plasma drift velocities and electron densities inferred from ground-based ionosonde measurements during low solar activity. J. Atmos. Sol. Terr. Phys..

[2] Huang X., Reinisch B.W. (1996). Vertical electron density profiles from the Digisonde ionograms: the average representative profile. Ann. De. Geophys..

[3] Adeniyi J.O., Adebesin B.O., Adimula I.A., Oladipo O.A., Olawepo A.O., Ikubanni S.O., Reinisch B.W. (2014). Comparison between African Equatorial Station Ground-based inferred vertical E x B drift, Jicamarca direct measured drift, and IRI model. Adv. Space Res..

[4] Adebesin B.O., Rabiu A.B., Adeniyi J.O., Amory-Mazaudier C. (2015). Nighttime morphology of vertical plasma drifts at Ouagadougou during different seasons and phases of sunspot cycles 20-22. J. Geophys. Res. – Space Phys..

