# Supplementary material for: F2-layer height of the peak electron density (hmF2) dataset employed in Inferring Vertical Plasma Drift – Data of Best fit
Source: Data Brief. 2018 May 5;19:59–66. doi: 10.1016/j.dib.2018.04.141 (PMC5992978; doi:10.1016/j.dib.2018.04.141)
Supplement: Supplementary file 1 — Supplementary material [file mmc1.docx]

**Conflict of interest**

There is no conflict of interest whatsoever with any individual, group, site, or agency regarding this data article.
